# Estrogen/ERR-α signaling axis is associated with fiber-type conversion of upper airway muscles in patients with obstructive sleep apnea hypopnea syndrome

**DOI:** 10.1038/srep27088

**Published:** 2016-06-02

**Authors:** H. H. Chen, J. Lu, Y. F. Guan, S. J. Li, T. T. Hu, Z. S. Xie, F. Wang, X. H. Peng, X. Liu, X. Xu, F. P. Zhao, B. L. Yu, X. P. Li

**Affiliations:** 1 Department of Otolaryngology-Head and Neck Surgery, Nanfang Hospital, Southern Medical University, Guangzhou, China; 2 Department of Neurobiology, Southern Medical University, Guangzhou, China; 3 Department of Anatamy, Southern Medical University, Guangzhou, China; 4 Department of Neurology, Nanfang Hospital, Southern Medical University, Guangzhou, China

## Abstract

Estrogen is related with the low morbidity associated with obstructive sleep apnea hypopnea syndrome (OSAS) in women, but the underlying mechanisms remain largely unknown. In this study, we examined the relationship between OSAS and estrogen related receptor-α (ERR-α). We found that the expression levels of ERR-α and Myh7 were both downregulated in palatopharyngeal tissues from OSAS patients. In addition, we report that ERR-α is dynamically expressed during differentiation of C2C12 myoblasts. Knockdown of ERR-α via instant siRNA resulted in reduced expression of Myh7, but not Myh4. Furthermore, differentiation of C2C12 cells under 3% chronic intermittent hypoxia, a model resembling human OSAS, was impaired and accompanied by a obvious reduction in Myh7 expression levels. Moreover, activation of ERR-α with 17β-estradiol (E2) increased the expression of Myh7, whereas pretreatment with the ERR-α antagonist XCT790 reversed the E2-induced slow fiber-type switch. A rat ovariectomy model also demonstrated the switch to fast fiber type. Collectively, our findings suggest that ERR-α is involved in estrogen-mediated OSAS by regulating Myhc-slow expression. The present study illustrates an important role of the estrogen/ERR-α axis in the pathogenesis of OSAS, and may represent an attractive therapeutic target, especially in postmenopausal women.

The prevalence of obstructive sleep apnea hypopnea syndrome (OSAS) in middle-aged individuals is 4% in males and 2% in females, with a strong male predominance^1^. Epidemiological studies have also shown that menopause is a significant risk factor for sleep apnea in women and that hormone replacement appears to be associated with reduced risk^2^,^3^. Estrogen may therefore play a protective role during OSAS pathogenesis, but the mechanisms by which estrogen contributes to OSAS remains largely unknown.

The most common pathogenic factors contributing to OSAS include anatomic abnormalities, obesity, and muscle dysfunction of the UA^4^. The function of mammalian skeletal muscle is determined by different muscle fiber types, which have variable actions in regards to the rate of force production, resistance to fatigue, and energy metabolism, and range from aerobic slow-twitch to anaerobic fast-twich physiology^5^,^6^. Our previous study demonstrated that the rate of type I slow-twitch myofiber (Myh7) was decreased in the palatopharyngeal muscles of OSAS patients^7^. Reduced rate of Myh7 can lead to fatigue and greater susceptibility to pharyngeal collapse during sleep^8^. In addition, skeletal muscle shows enormous plasticity, even in adults, in response to a variety of stimuli, such as exercise^9^, environmental factors^10^, nerve signals, hypoxia, and hormones^11^.

As a transcription factor, ERR-α plays roles in mitochondrial metabolism, energy substrate uptake, and biogenesis^12^. Moreover, ERR-α appears to be required for normal skeletal myocyte differentiation and may exert its effects via modulation of MAP kinase signaling^13^. However, it is unclear whether ERR-α mediates the conversion of fiber types of UA muscle in OSAS patients or contributes to pharyngeal collapse.

In this study, we investigated the relationship between ERR-α and OSAS by examining the expression of ERR-α and Myh7 in male patients with OSAS. Furthermore, we also explored the the possible mechanisms underlying fiber-type switch in OSAS patients. A clearer understanding of how sex hormones influence the morbidity associated with OSAS could enable therapeutic interventions for high-risk female patients experiencing postmenopause.

## Results

### Expression levels of ERR-α and Myh7 are decreased in palatopharyngeal tissues from OSAS patients

**Figure 1:**
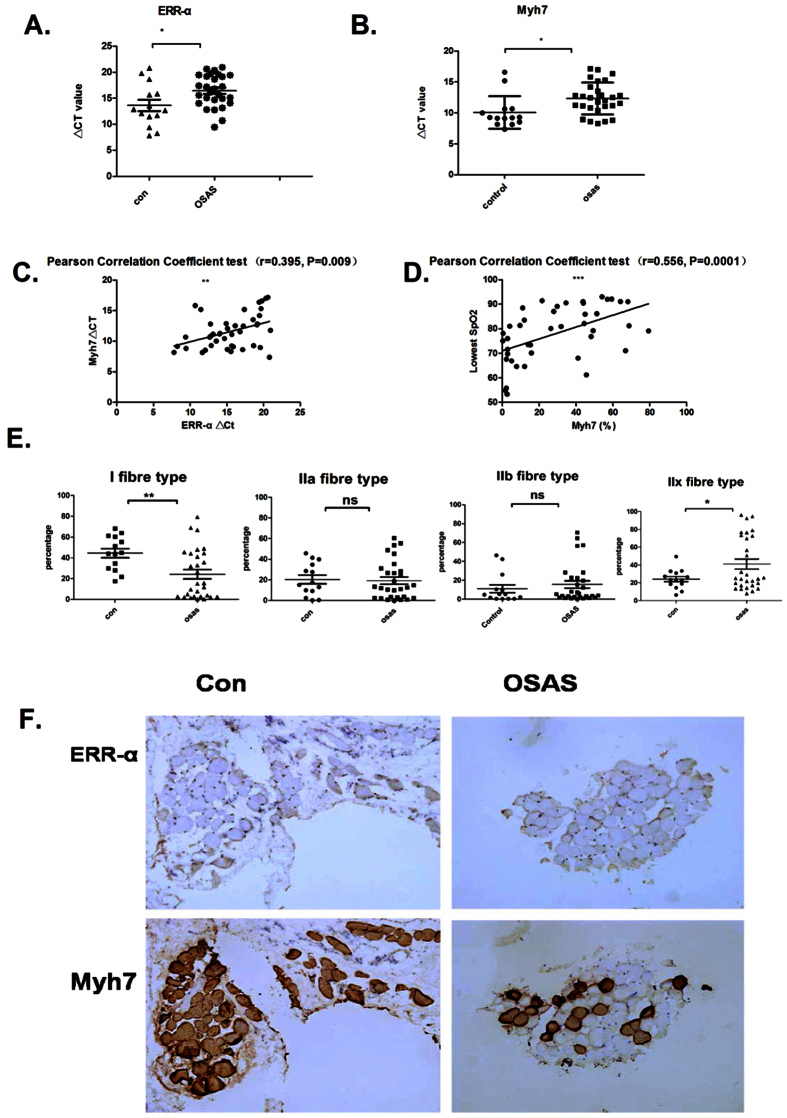
ERR-α and Myh7 levels are decreased in OSAS palatopharyngeal tissues. (**a**) qRT-PCR analysis of ERR-α mRNA levels. The ΔCT value of ERR-α was significantly different between OSAS and control tissues (t = 2.494, p < 0.05). (**b**) qRT-PCR analysis of Myh7 mRNA levels. The ΔCT value of Myh7 was significantly different between OSAS and control tissues (t = 2.658, p < 0.05). (**c**) Pearson’s correlation coefficient was calculated to test the correlation between ERR-α and Myh7 expression. A significant positive correlation was observed (n = 42 specimens, r = 0.395, p < 0.01). (**d**) Pearson’s correlation coefficient was calculated to test the correlation between Spo2 and Myh7 expression. A significant positive correlation was observed between the percentage of Myh7 and the lowest SPO_2_ (n = 42 specimens, r = 0.556, p = 0.0001). (**e**) qRT-PCR analysis of the expression levels of different muscle fiber types. Significant differences were observed between the OSAS and control groups for type I (p < 0.05) and type IIx fibers (p < 0.05). (**f**) Representative images of immunohistochemical staining for ERR-α and Myh7 in palatopharyngeal tissues. Magnification = 200x. Dark blue-brown staining represents positive ERR-αantigen. Statistical analysis were performed using independent t tests (A, B, E) and Pearson Correlation test (C, D). *p < 0.05, **p < 0.01, ***p < 0.001, *p < 0.05, **p < 0.01, ***p < 0.001.

We performed real-time PCR (qRT-PCR) to assess mRNA levels of ERR-α and Myh7 in palatopharyngeal tissues isolated from patients with OSAS or patients with chronic tonsillitis (control). We examined polysomnographic data, ERR-α and Myh7 expression levels, and the percentages of Myh7 (fibre type I), Myh2 (fibre type IIa), Myh1(fibre type IIx), and Myh4 (fibre type IIb) in palatopharyngeal tissues from control and OSAS patients. All of the factors except age, shown in Table 1, have significant differences between OSAS and control group. We found that ERR-α and Myh7 were both downregulated in tissues from OSAS patients compared to tissues from control patients (Fig. 1a,b). We investigated the relationship between expression levels of ERR-α and Myh7 using Pearson’s correlation coefficient and found a significant positive association (r = 0.395, p < 0.01; Fig. 1c). Interestingly, the lowest Spo_2_ (oxygen saturation) was also positively correlated with Myh7 (r = 0.556, p < 0.01; Fig. 1d). Moreover, we observed that fiber type IIx was increased, whereas fiber type I was decreased in tissues from OSAS patients compared with those from control patients (Fig. 1e). Further immunohistochemical staining for ERR-α and Myh7 in the palatopharyngeal tissues confirmed a similar trend in protein levels (Fig. 1f). Taken together, these data suggest that ERR-α expression is decreased in OSAS and is closely associated with the expression of Myh7. Therefore, ER/ERR-α signaling may be involved in the regulation of fiber-type conversion in OSAS patients.

### ERR-α is required for proper C2C12 differentiation and expression of Myh7 in myoblasts and myotubes

**Figure 2:**
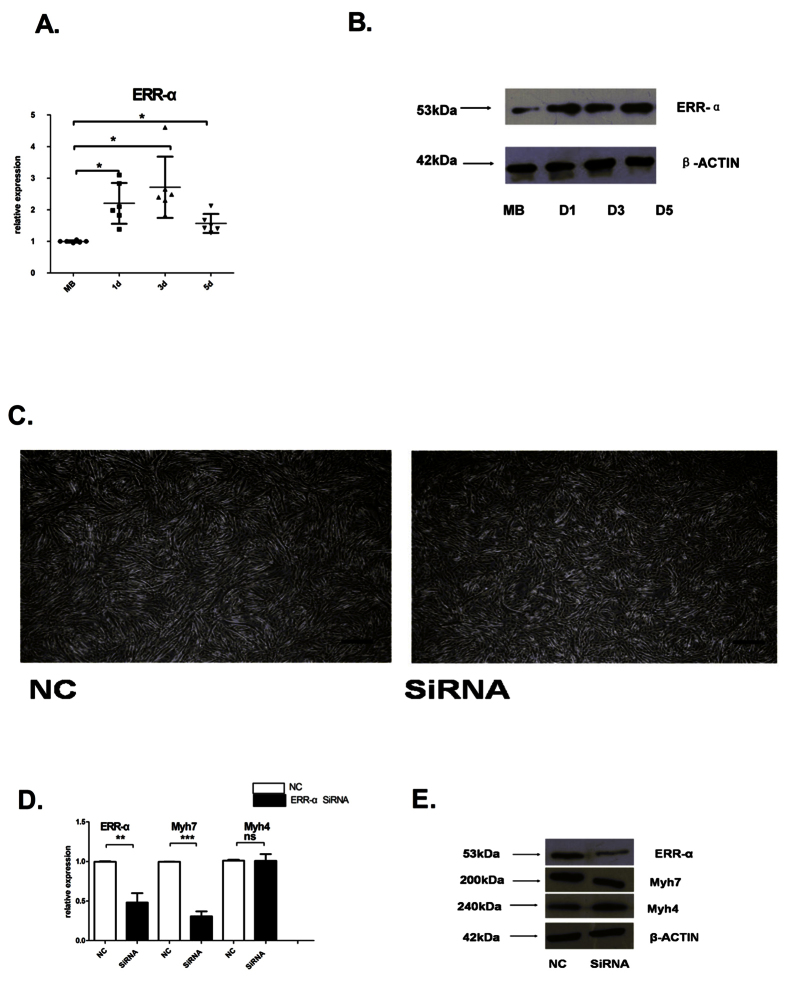
ERR-α and Myh7 expression patterns during C2C12 differentiation. (**a**) qRT-PCR analysis of ERR-α levels relative to 18S levels at indicated time points during MT differentiation. Graphs represent fold change over the expression level at MB stage. Results shown are representative of at least six independent experiments. (**b**) Western blot analysis of ERR-α protein levels during C2C12 differentiation. β-actin was used as a loading control. MB represented the control group. (**c**) Representative images at 4 h C2C12 differentiation after siRNA transfection. Magnification = 40x, scale bar = 500 μm. (**d**) qRT-PCR analysis of ERR-α, Myh7, and Myh4 levels relative to 18S levels in C2C12 cells transfected with ERR-α siRNA compared with control siRNA (NC). Graph represents fold change over the expression level of NC. Results shown are representative of at least three independent experiments. (**e**) Western blot analysis of ERR-α, Myh7, and Myh4 protein levels. β-actin was used as a loading control. Statistical analysis in (**a**) was performed using one-way ANOVA and Dunnett’s T3 test and in (**d**) using independent t tests. *p < 0.05, **p < 0.01, ***p < 0.001.

Recently, many studies have focused on the role of ERR-α and its coactivator PGC-1α in the direct transcriptional regulation of genes involved in energy metabolism^14^. We compared the relative expression of ERR-α in day (d) 0 myoblast (MB) and d1, d3, and d5 myotube (MT) by qRT-PCR (Fig. 2a). We found that ERR-α was dynamically expressed (1.57-fold to 2.71-fold in MT) during C2C12 differentiation. A similar trend was observed for ERR-α protein levels (Fig. 2b). Our findings are consistent with a recently reported transcript expression profilefor ERR-α in C2C12 cells^13^.

To examine the contribution of ERR-α to MB differentiation, we used small interfering RNA (siRNA) to knockdown ERR-α in C2C12 cells. SiRNA-mediated downregulation of ERR-α (0.48-fold) resulted in reduced MT formation and was associated with significantly decreased mRNA (0.31-fold) and protein levels of Myh7 (Fig. 2c–e). However, ERR-α-depletion had no effect on the expression of Myh4. These results suggest that ERR-α affects the onset and maintenance of Myh7 expression during skeletal myocyte differentiation.

### Chronic intermittent hypoxia inhibits the expression of Myh7at all stages of C2C12 differentiation, but inhibits ERR-α only during early stages

**Figure 3:**
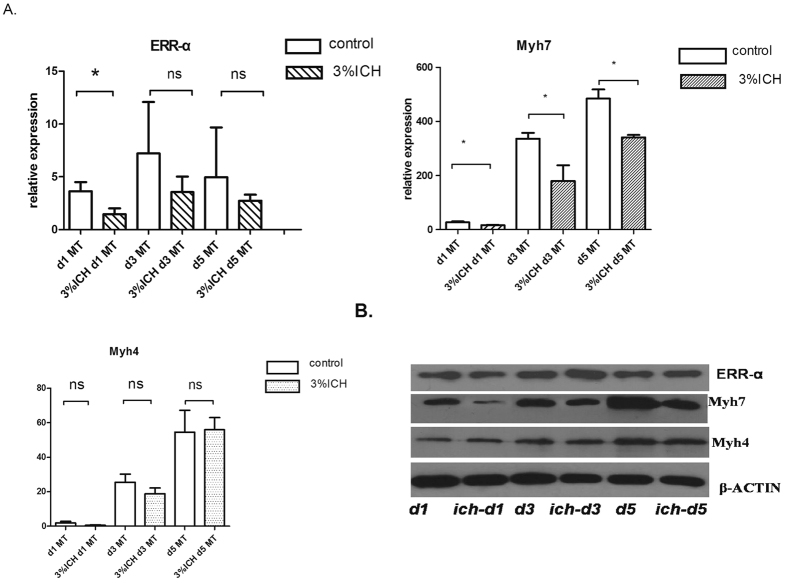
Myh7 and ERR-α expression levels are suppressed by CIH. (**a**) qRT-PCR analysis of ERR-α,Myh7, and Myh4 levels relative to 18S in C2C12 cells exposed to 3% chronic intermittence hypoxia (CIH) or 21% O_2_(control) over the course of differentiation. Results shown are representative of at least three independent experiments. (**b**) Western blot analysis of ERR-α, Myh7, and Myh4 protein levels in C2C12 cells exposed to 3% CIH or 21% O_2_. Normal differentiation cell were as control group. β-actin was used as a loading control. Statistical analysis in (**a**) was performed using independent t tests. *p < 0.05, **p < 0.01, ***p < 0.001.

Chronic intermittent hypoxia (CIH) is a characteristic pathological component of OSAS^15^. To recapitulate human OSAS pathology *in vitro*, we exposed C2C12 cells to 3% CIH^16^. C2C12 cells exposed to CIH only moderately underwent MT fusion and displayed reduced Myh7 levels during all stages of differentiation compared to cells under normoxia (d1MT, 16.573 ± 1.407 vs. 26.852 ± 3.881; d3MT, 180.14 ± 57.75 vs. 335.72 ± 21.93; d5MT, 341.18 ± 8.97 vs. 484.33 ± 33.39). Myh4 protein levels increased over the course of C2C12 differentiation, but were not significantly affected by CIH (Fig. 3a,b). Furthermore, comparing with normoxia, ERR-α levels decreased only during the early stages (d1MT) of differentiation under CIH (Fig. 3a,b).

### Estrogen stimulation promotes Myh7 expression, but is counter acted by an ERR-α antagonist

**Figure 4:**
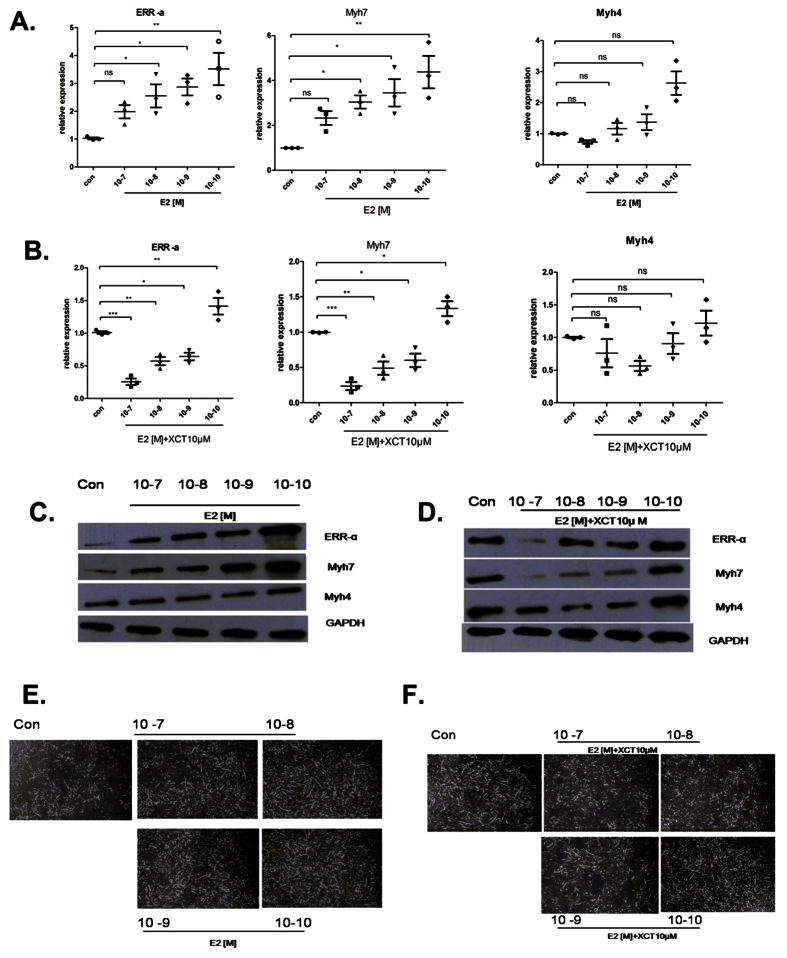
Effects of estrogen and ERR-α antagonists on ERR-α and Myh7 expression levels. (**a**) qRT-PCR analysis of ERR-α, Myh7, and Myh4 levels relative to 18S in C2C12 cells pretreated with 10^−7^–10^−10^ M estrogen (E2) for 48 h (d2MT) compared with control cells (con). (**b**) qRT-PCR analysis of ERR-α, Myh7, and Myh4 levels relative to 18S in C2C12 cells 48 h (d2MT) after differentiation pretreated with 10^−7^–10^−10^ M estrogen (E2) and 10 μM XCT790 compared with control cells (con). Results shown are representative of at least three independent experiments. (**c**) Western blot analysis of ERR-α, Myh7, and Myh4 protein levels in d2 C2C12 cells pretreated with 10^−7^–10^−10^ M estrogen (E2) compared with control cells (con). GAPDH was used as a loading control. **(d**) Western blot analysis of ERR-α,Myh7, and Myh4 protein levels in d2MT C2C12 cells pretreated with 10^−7^–10^−10^ M estrogen (E2) and 10 μM XCT790 compared with control cells (con). GAPDH was used as a loading control. Statistical analysis was performed using one-way ANOVA and Dunnett’s T3 test. *p < 0.05, **p < 0.01, ***p < 0.001. (**e**) Representative images of C2C12 cells pretreated with 10^−7^–10^−10^ M estrogen (E2) at d2MT of differentiation. Magnification = 40x, scale bar = 500 μm. (**f**) Representative images of C2C12 cells pretreated with 10^−7^–10^−10^ M E2 and 10 μM XCT790 at d2MT of differentiation. Magnification = 40x, scale bar = 500 μm.

We found that endogenous ERR-α expression is induced during myogenesis. Therefore, we assessed whether ERR-α overexpression, stimulated by estrogen, affected MT formation. The addition of 17β-estradiol (E2) during C2C12 differentiation significantly induced MT formation (Fig. 4e). The qRT-PCR analysis showed that ERR-α obviously increased (1.98–3.52-fold) in response to E2. The expression of Myh7 also increased (2.33–4.38-fold; Fig. 4a) in response to E2, but there was not statistically significant for Myh4 (0.73—2.63-fold; Fig. 4a). We also observed a similar trend in protein expression changes (Fig. 4a).

We evaluated whether the effect of E2 could be blocked by XCT790, a specific antagonist of ERR-α^13^. Pretreatment of C2C12 cells with XCT790 and E2 caused a delay in MT formation (Fig. 4f). Moreover, XCT790 blocked the E2-stimulated increase in ERR-α and Myh7 mRNA (Fig. 4b) and protein levels (Fig. 4d) observed in d2 MT. Importantly, Myh4 expression was unaffected by E2 or XCT790. Collectively, these results suggest that the ER/ERR-α axis regulates Myh7 expression during myogenesis.

### ERR-α expression and Myh7 are decreased in sternohyoid muscles of ovariectomized rats

**Figure 5:**
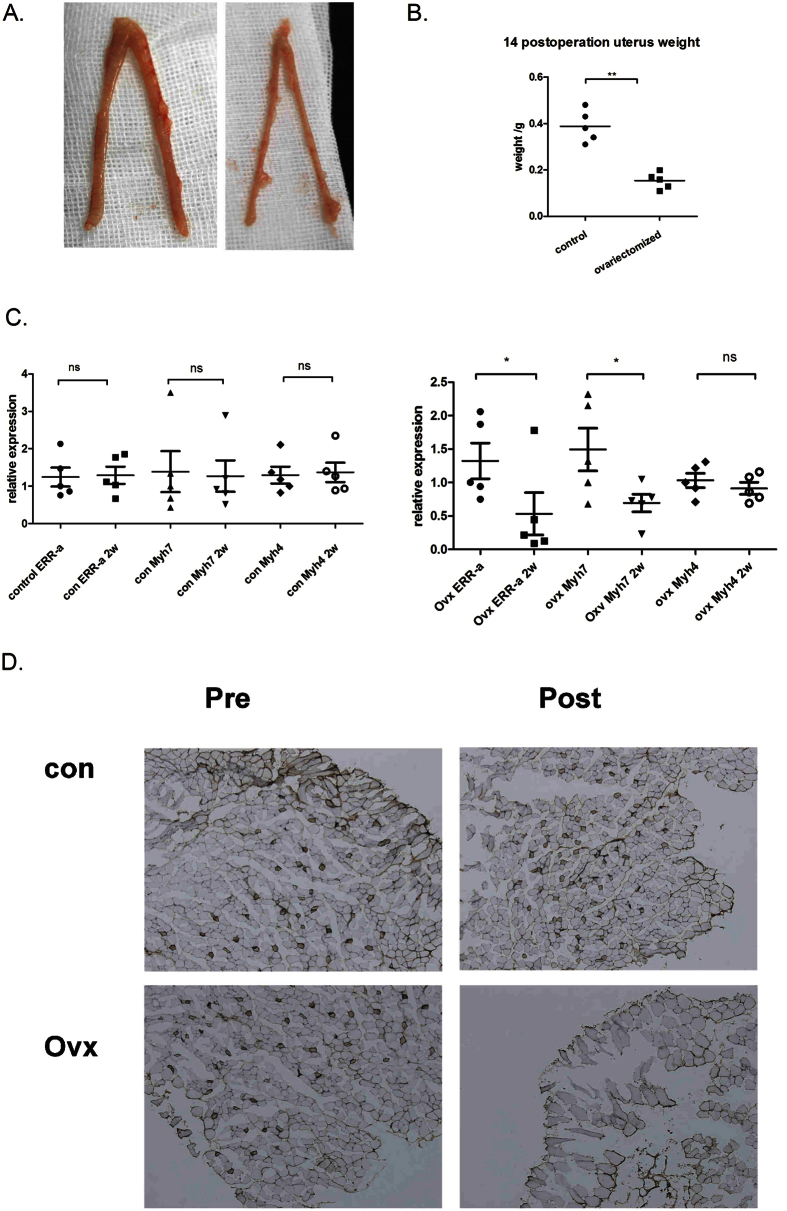
Effects of ovariectomy on ERR-α and Myh7 expression levels. (**a**) Marked uterine hypoplasia was obvious in ovariectomized (OVX) but not sham rats 2 weeks after surgery. (**b**) Weight of uteri 2weeks after OVX or sham surgery. (**c**) qRT-PCR analysis of ERR-α, Myh7, and Myh4 levels relative to 18S in sternohyoid muscles before and 2 weeks after surgery. (**d**) Representative images of immunohistochemical staining for Myh7 in sternohyoid muscle before and 2 weeks after surgery. Magnification = 100x. Data are presented as means ± SEM. Statistical analysis was performed by independent t test or paired t test. *p < 0.05, **p < 0.01, ***p < 0.001, OVX vs. sham. Similar results were observed in three independent experiments.

E2-induced regulation of ERR-α and Myh7 expression in C2C12 cells prompted us to investigate the existence of a similar mechanism *in vivo*. To address this question, we used ovariectomized (OVX) rats, which recapitulate the changes observed during human postmenopause. Marked hypoplasia in rats that underwent OVX was confirmed by visual inspection (Fig. 5a) and by measuring the weight of the dissected uterus two weeks after surgery (OVX, 0.15 g vs. sham, 0.39 g; Fig. 5b). RNA was extracted from the sternohyoid muscle before and two weeks after surgery. The qRT-PCR analysis revealed that ERR-α (0.40-fold) and Myh7 (0.46-fold) expression levels were significantly decreased two weeks after OVX (Fig. 5c). But Myh4 (0.83-fold) expression had no significantly change 2wk after OVX (Fig. 5c). Immunohistochemical staining of sternohyoideus muscle revealed obviously decreased Myh7 protein levels two weeks after OVX comparing with pre-operation (Fig. 5d). These data suggest that OVX is associated with decreased Myh7 and ERR-α expression levels *in vivo*.

## Discussion

The clinical data presented in this study (Fig. 1) strongly suggest that ERR-α plays a role in the regulation of fiber-type conversion in OSAS patients, a characteristic of OSAS pathophysiology^8^. Additionally, differences in the expression of Myhc-slow (Myh7) did not appear to be attributed to differences in age. Alterations in fibre type and MyHC composition are suggestive of a neuromuscular disorder of the soft palate in SDB patients^17^. These changes may ultimately impair the ability of the muscles to maintain pharyngeal patency. For the first time, we report that Myh7 was positively correlated with ERR-α and some parameters in the PSG report. Most studies suggest that OSAS is associated with increased type II and decreased type I fibers. However, our findings show that OSAS is associated with increased type IIx (Myh1), rather than the whole type II fibers, and decreased type I fibers in palatopharyngeal tissues. It remains uncertain if such alterations in fiber type directly cause OSAS^17^. The actual fiber-type changes underlying OSAS are far from clear and are likely to be associated with many factors, such as age, gender, and biopsy position.

ERR-α is an important transcription factor that regulates oxidative stress and metabolic homeostasis. The ERR-α gene is stimulated by estrogen in the mouse uterus and heart, but not in the liver^18^. The ERR-α/PGC-1α axis regulates the expression of genes involved in glucose oxidation, left ventricular systolic function, and heart failure^19^. PGC-1α is a principal factor that regulates muscle fiber-type determination^20^. Therefore, estrogen may increase the endurance of muscle by regulating muscle fiber type through the ERR-α pathway. In particular, special attention should be given to functional muscle alterations of the airways, as these may influence the evolution and treatment of the OSAS^21^.

We also observed that ERR-α was dynamically expressed during C2C12 differentiation and affected the expression levels of Myhc-slow (Myh7), but not Myhc-fast (Myh4). Furthermore, 3% CIH inhibited the expression of Myhc-slow, but not Myhc-fast, during the entire course of differentiation, and inhibited ERR-α only at early stages of differentiation. CIH is a hallmark of OSAS thatinitiates a cascade of events involving oxidative stress and inflammatory processes^22^. Episodic hypoxia, a consequence of periodic airway occlusion, is responsible for OSAS progression through the impairment of the neural control systems and through altered respiratory muscle contractile function, leading to a vicious cycle of further airway obstruction^23^. Many studies utilize CIH as the exposure factor underlying OSAS^16^,^24^. CIH increased the diaphragm muscle type IIb (Myh4) areal density in adult male Wistar rats^25^. Our CIH experiments demonstrate that ERR-α may participate in the early stages of fiber-type switch. Moreover, estrogen improved the endurance of the genioglossus muscle in OVX rats exposed to CIH^26^.

Female patients experiencing postmenopause or exhibiting a higher BMI present with OSAS more frequently than their male counterparts^27^. Estrogen could influence exercise metabolism^28^ and pharyngeal dilator muscle activity^29^. A significant increase in genioglossus electromyogram (EMGgg) was found in the postmenopausal group re-examined after hormone therapy^29^,^30^. Denies and colleagues investigated the long-term effects of a high-fat diet and subsequent obesity on muscle fiber type composition and found that the high-fat diet was associated with a significantly lower proportion of slow, type I fibers in the soleus muscle of male, but not female, mice^31^. The critical pathophysiological feature of OSAS is sleep-related pharyngeal collapse of the UA, which is partly determined by the properties of different muscle fiber types. The underlying mechanisms of low morbidity associated with OSAS in female patients are not completely understood. Hormone replacement therapy is still not widely used for the prevention and treatment to these postmenopausal women with high risk for OSAS. Therefore, further investigation is urgently needed.

Lastly, our data suggest that E2 regulates Myhc-slow expression in a concentration-dependent manner. The inhibition of estrogen signaling by the ERR-α antagonist XCT790 was able to completely abolish E2-induced expression of Myhc-slow. Furthermore, animal experiments also demonstrated that ERR-α and Myhc-slow were decreased in the OVX group. These findings suggest that ERR-α expression is regulated by estrogen in C2C12 cells *in vitro* and in muscles *in vivo*. Taken together, the data imply that ERR-α intersects with the estrogen axis in muscle to promote MT formation and shift fiber types from Myhc-fast to Myhc-slow.

## Conclusions

We have demonstrated for the first time that ERR-α expression is markedly reduced in OSAS patients. E2 can upregulate Myhc-slow expression in a concentration-dependent manner through ERR-α, which maybe contribute to improve UA muscle fatigue. Multiple lines of evidence suggest that ERR-α is involved in E2-mediated low morbidity by regulating Myhc-slow expression. These findings highlight ERR-α/Myhc-slow as an important transcriptional regulatory cascade contributing to E2-mediated muscle protection, thus providing a potentially novel therapeutic target for the treatment of postmenopausal OSAS.

## Methods

### Clinical specimens

Primary biopsy specimens and normal biopsies of palatopharyngeal muscle were obtained from the Otolaryngological Department in Nanfang Hospital (Southern Medical University, Guangzhou, China). Twenty-eight adult male patients with severe OSAS and 14 adult male patients with chronic tonsillitis, but not OSAS (control group), were recruited between February 2011 and July 2013. Both tissue types were histologically confirmed by hematoxylin and eosin (H&E) staining. No patient reported a history of local operation, radiotherapy, or neuromuscular disease. Informed consent was obtained from each patient, and the research protocols were approved by the Ethics Committee of Nanfang Hospital. Methods were carried out in accordance with the guidelines of Ethics Committee of Nanfang Hospital. The project was approved by Chinese ClinicalTrials. gov (ChiCTR-CCC-13003415) on May 31, 2013. Diagnoses and surgical treatments for all OSAS patients were performed according to the guidelines established by the Chinese Medical Association. Disease severity was assessed by polysomnography(PSG). Sleep apnea was defined as airflow being reduced to less than 10% of baseline for more than 10 s. Hypopnoea was defined as airflow being reduced to less than 30% of baseline for more than 10 s in association with a 4% oxygen desaturation or less than 50% of baseline for more than 10 s in association with a 3% oxygen desaturation or micro-arousal. When AHI was >30, we defined the condition as severe.

### Cell culture and reagents

Mouse C2C12 myoblasts (ATCC) were cultured in high glucose DMEM containing10% fetal bovine serum (FBS) and 1% penicillin-streptomycin antibiotic solution. The differentiation media contained 2% horse serum instead of 10% FBS. Cells were passaged by trypsinization (0.5% trypsin in 0.5 mM EDTA, Gibco BRL) upon reaching 70% confluency. Normoxic cells were cultured under a humidified atmosphere of 95% air and 5% CO_2_ at 37 °C. For hypoxia experiments, cells were exposed to 3% O_2_ in an incubated chamber (Thermo 3111; USA) adjusted to the desired O_2_ concentration. Episodic cycles were set as follows: 8 h per day of 3% O_2_, 5% CO_2_, and balanced N_2_ for 35 min, then 21% O_2_ for 25 min, followed by 16 h of 21% O_2_ over the course of 5 days^16^. To investigate the effects of 17β-estradiol (E2) on differentiation, cells were seeded in 6-well plates (Corning, Falcon) in the presence or absence of E2 (Sigma, USA). E2 diluted in ethanol was added to cells to a final concentration of 10^−7^ to 10^−10^ M at the beginning of differentiation and cells were collected after 48 h. XCT790 (Sigma, USA) was diluted in DMSO and added to cells to a final concentration of 10 μM, the same volume of DMSO as blank control.

### siRNA transient transfection of C2C12 cells

We purchased siRNA oligonucleotides for ERR-α from Invitrogen™ Life Technologies. Cells were transfected with negative control (NC) oligos or ERR-α-specific oligos for 48 h. Transfections were performed withLipofectamine 2000 (Invitrogen, Carlsbad, USA), according to the manufacturer’s directions, using 100 nMsiRNA per well of a 6-well plate. Cells were cultured in complete media without antibiotics until they reached 50–60% confluency 24 h after plating and were then incubated with siRNA-liposome complexes. Cells were harvested 48 h and 72 h later for Western blot and qRT-PCR assays.

### RNA extraction and qRT-PCR

Total RNA was extracted from the samples using RNAiso Plus (TAKARA, Shiga, Japan), according to the manufacturer’s instructions. RNA concentration was measured by spectrophotometry. After cDNA synthesis (All-in-One First-Strand cDNA Synthesis kit, GeneCopoeia Inc., USA), qRT-PCR was performed using All-in-One qPCR Mix (GeneCopoeia Inc., USA) on the ABI 7500HT System (Applied Biosystems) with primers described in Table 2. PCR conditions were as follows: 95 °C for 10 min followed by 40 cycles of 95 °C for 10 s, 60 °C for 20 s, and 72 °C for 34 s. The specificity of each qRT-PCR reaction was verified by melting curve analysis. Duplicate reactions were run for each sample. All samples were normalized based on the expression levels of 18S ribosomal RNA and fold changes were calculated using the 2^ΔΔCT^ method. The percentage of MyhX in the sample was calculated as follows (where X = 1, 2, 4, or 7):


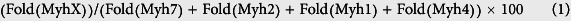


### Western blot analysis

C2C12 cells were harvested and extracted with lysis buffer (Genechem, Shanghai, China) at 4 °C. Nuclear protein extracts were prepared using a protein fractionation kit (Genechem, Shanghai, China), according to the manufacturer’s directions. Total protein was isolated and quantified using the BCA assay. The protein lysates were separated on a 10% SDS-PAGE gel and then transferred to polyvinylidenedifluoride (PVDF) membranes (Millipore, USA). Primary antibodies were diluted in 5% non-fat milk prepared in phosphate-buffered saline (PBS) containing 0.05% Tween-20 and applied to the membranes with gentle shaking overnight at 4 °C. The next day, membranes were incubated with horseradish peroxidase (HRP)-labeled goat-anti mouse IgG (1:5000; Santa Cruz Biotechnology) for 60 min at room temperature and then washed in 0.05% Tween-20/PBS for 4 min. Finally, X-ray film was exposed to the PVDF membranes for 1 min. The intensity of the chemiluminescent signals was quantified using Image J software. The ERR-α antibody was purchased from Millipore (USA), the Myh4 antibody was purchased from Proteintech Group Inc (USA), and the Myh7 antibody was purchased from Abcam (USA). Beta-actin or GAPDH were used as internal standards to control for protein loading.

### Immunohistochemistry

Formalin-fixed, paraffin-embedded muscle tissue samples were sectioned to 4 μm thickness and stained with ERR-α antibody (1:125; Millipore, USA) and Myh7 antibody (1:400; Abcam, USA).

### Ovariectomy of rats

Age-matched female Sprague-Dawley(SD) rats (permission number: (SCXK(Yue)2011–0029) weighing 180–200 g at the time of arrival were used for all experiments. Each group of five SD rats was kept under standard laboratory conditions for up to 2 weeks. Ovariectomy (OVX) or sham operations (sham) were conducted after successful general paralysis. Sternohyoid muscle biopsies were collected before and 2 weeks after OVX or sham surgery. Animals were sacrificed and the uteri weighed to ensure efficacy of the OVX surgery. Total RNA from sternohyoid muscle samples, which had been snapfrozen in liquid nitrogen, was isolated using Trizol reagent (Life Technologies). Samples were then analyzed by qRT-PCR and immune histochemistry as described above. All experiments were performed in accordance with the Animal Facility Institutional Animal Care and Use Committee regulations. All experimental protocol on the animals were approved by Nanfang Hospital Animal Care Committee (NFHACC 2011-CZ102).

### Statistical analysis

SPSS 13.0 for windows software was used for statistical analysis. Data are presented as mean ± SEM. All experiments were performed at least three independent times. The onesample Kolmogorov-Smirnov test was used to analyzenormally distributed data. Levene’stest was used to assess homogeneityofvariance. The two-tailed Student’s t test was used for comparisons between two independent groups. One-way ANOVA and Dunnett’s T3 tests were used for comparisons between multiple independent groups. P values of <0.05 were considered statistically significant.

## Additional Information

**How to cite this article**: Chen, H. H. *et al*. Estrogen/ERR-α signaling axis is associated with fiber-type conversion of upper airway muscles in patients with obstructive sleep apnea hypopnea syndrome. *Sci. Rep.* **6**, 27088; doi: 10.1038/srep27088 (2016).

## Figures and Tables

**Table 1:** Comparison of polysomnography data, ERR-α and Myh7 relative expression, and percentage of Myh7 in palatopharyngeal tissues between control and OSAS groups (two independent t-test).

**Parameter**	**Con (n = 14)**	**OSAS (n = 28)**	**95% CI**	***P***
Age	37.43 ± 8.13	40.32 ± 6.78	−7.69--- 1.90	0.23
AHI	1.46 ± 1.41	59.21 ± 22.47	−66.48--- −48.99	0.00*
ERR-α△CT	13.62 ± 4.08	16.42 ± 3.06	−5.07--- −0.53	0.017*
Myh7△CT	10.23 ± 2.56	12.33 ± 2.55	−3.79--- −0.41	0.016*
Myh7%	43.68 ± 16.85	21.97 ± 23.88	36.16--- 7.26	0.004*
BMI	22.89 ± 2.20	28.23 ± 3.00	−7.17--- −3.51	<0.005*
Lowest Spo2	89.93 ± 2.27	72.16 ± 8.67	14.22–21.33	<0.005*
Average Spo2	95.79 ± 0.65	92.68 ± 3.71	1.64–4.59	<0.005*

**Table 2:** Primers for qRT-PC.

**Primer**	**Description**
Human Myh7	Accession No: NM000257.2, product length: 112
	Sense 5′-TGCCACATCTTGATCTGCTC-3′;
	Antisense 5′-CTCGGCTTCAAGGAAAATTG-3'
Human ERR-α	Accession No: NM-004451.3, product length 116
	Sense 5′-CACTATGGTGTGGCATCCTGT-3'
	Antisense 5′-CGTCTCCGCTTGGTGATCTC-3'
Human Myh4	Accession No: NM-017533.2, product length: 86
	Sense 5′- CCCGCAATGCAGAGGAGAA -3'
	Antisense 5′- GCTGGTGTCCTGTTCCTTCTTC -3'
Human Myh2	
	Accession No: NM017534.5, product length: 109
	Sense 5′-CTGATGCCATGGAATGACTG-3'
	Antisense 5′-CCCTATGCTTTATTTCCTTTGC-3
Human Myh1	Accession No: NM-005963.3, product length: 98
	Sense 5′- GGAGGAACAATCCAACGTCAA -3'
	Antisense 5′- TGACCTGGGACTCAGCAATG -3'
Human 18S	

	Sense 5′-GTAACCCGTTGAACCCCATT-3'
	Antisense 5′-CCATCCAATCGGTAGTAGCG-3'
Rat Myh7	
	Accession No: NM-17240.2, product length: 159
	Sense 5′-TACAATGCGCAAGTGGTAGC-3'
	Antisense 5′- GACGGTCTTACCAGCTCCG-3'
Rat Myh4	Accession No: NM-019325.1, product length:136
	Sense 5′-CAG GTC AAC AAG CTG -3'
	Antisense 5′-GAT ATA CAG GAC AGT GAC AAA GAA CG-3'
Rat ERR-α	Accession No: Nm-001008511.2, product length: 301
	Sense 5′-AGTACAGCTGTCCGGCCTCCAAC-3′
	Antisense 5′-GGCATGGCATACAGCTTCTCAGG -3'
Rat 18S	
	Sense 5′- GGGAGGTAGTGACGAAAAATAACAAT-3'
	Antisense 5′- TTGCCCTCCAATGGATCCT-3
